# Sustainable Control of Carbon Emissions and Energy Consumption Through a Green Data Center Approach

**DOI:** 10.1155/tswj/1158756

**Published:** 2026-05-20

**Authors:** Yasir Afzal, Naila Nawaz, Abdullah Ayub Khan, Muhammad Jawad Yousaf, Muhammad Zohaib Khan, Erssa Arif, Muqaddas Salahuddin, Mohamad Afendee Mohamed, Sajid Ullah

**Affiliations:** ^1^ Faculty of Computer Science, Riphah International University, Faisalabad, Pakistan, riphah.edu.pk; ^2^ Department of Computer Science, NUML Faisalabad Campus, Faisalabad, Pakistan; ^3^ Kohat University of Science and Technology, Kohat, Pakistan, kust.edu.pk; ^4^ Department of Computer Science, Bahria University Karachi Campus, Karachi, Pakistan, bahria.edu.pk/; ^5^ Faculty of Informatics and Computing, Universiti Sultan Zainal Abidin, Besut Campus, Besut, Malaysia, unisza.edu.my; ^6^ Department of Information Technology, Shaheed Mohtarma Benazir Bhutto Institute of Trauma, Karachi, Pakistan; ^7^ Faculty of Computer Science and Information Technology, Superior University, Lahore, Pakistan, superior.edu.pk; ^8^ Department of Water Resources and Environmental Engineering, Nangarhar University, Jalalabad, Nangarhar, Afghanistan

**Keywords:** data center (DC), energy efficiency (EE), energy optimization (EO), genetic algorithm (GA), physical machines (PMs), virtual machine (VM)

## Abstract

Data center management, the foundation of contemporary cloud computing, has made energy saving a top priority. Among other difficulties, the placement of virtual machines (VMs) has a major impact on data center resource and energy usage. Assigning VMs to physical machines (PMs) is a challenging NP‐hard problem, especially in large‐scale infrastructures where it is computationally infeasible to find an ideal solution. To solve the VM placement problem, the proposed study formulates it as a restricted optimization problem with the goal of preserving performance while lowering energy consumption. The explosive growth of data centers has resulted in higher energy consumption and higher carbon dioxide (CO_2_) emissions, which are a primary cause of climate change. Globally, governments, energy‐focused organizations, and business executives have taken notice of this expanding environmental impact. This study provides a comprehensive analysis of data center energy consumption patterns, environmental effects, and trends in energy consumption. It also suggests doable energy‐saving measures, such as installing energy‐efficient infrastructure and upgrading air conditioning systems. The paper also presents an improved genetic algorithm–based method that is tailored for energy‐conscious VM deployment, successfully striking a balance between computing economy and convergence accuracy. The suggested solution highly increased data centers′ energy efficiency by incorporating this strategy within a profile‐based virtual resource management model. Additionally, policy suggestions for sustainable data center management are delineated, advancing the more general objective of ecologically conscious cloud computing. Experimental results demonstrate that the proposed method achieves up to 50% reduction in execution time, 48% fewer generations for convergence, and approximately 7% reduction in energy consumption compared to traditional first fit decreasing (FFD) methods. Additionally, the integration of task classification improves energy efficiency by up to 15% and reduces the number of active PMs. These findings highlight the effectiveness of the proposed framework in enabling scalable, energy‐efficient, and environmentally sustainable cloud data center management.

## 1. Introduction

Cloud computing is a widely used distributed architecture that provides scalable, on‐demand services through the Internet by shifting data and processing to large‐scale data centers (DCs). It supports sectors such as healthcare, finance, enterprise, and transportation. The rapid growth of DCs driven by Internet of Things (IoT), Big Data, and AI has improved service delivery but also increased environmental concerns. However, there are significant environmental issues associated with this increase. Due to their heavy reliance on fossil fuels, DCs release around 43 million tons of CO_2_ yearly, an amount that is increasing by 11% annually and was expected to contribute to 5.5% of global carbon emissions since 2023. Their energy consumption was projected to surpass that of the major economies by 2026, reaching 10,000 TWh. ICT equipment is powered and cooled by more than 93% of this energy; one DC uses as much electricity as 25,000 households. Although renewable energy provides some respite, its intermittent nature limits reliability. Thus, to lower consumption and guarantee a sustainable future for cloud computing, integrated resource management and energy‐efficient techniques are essential. Recent studies have focused on the integration of machine learning with cloud‐based optimization techniques for efficient resource allocation and carbon‐aware computation. The goal of cloud computing is to give consumers immediate, on‐demand access to computer resources so that service delivery can be flexible and scalable. DCs and other underlying infrastructure, especially physical equipment, must expand in tandem with the number of users. Energy conservation is a significant obstacle to this expansion, however. Growing energy consumption is directly correlated with the expansion of physical machines (PMs). One important tactic for reducing energy usage without sacrificing performance on PMs is to strategically place virtual machines (VMs) [1, 2].

**Figure 1:**
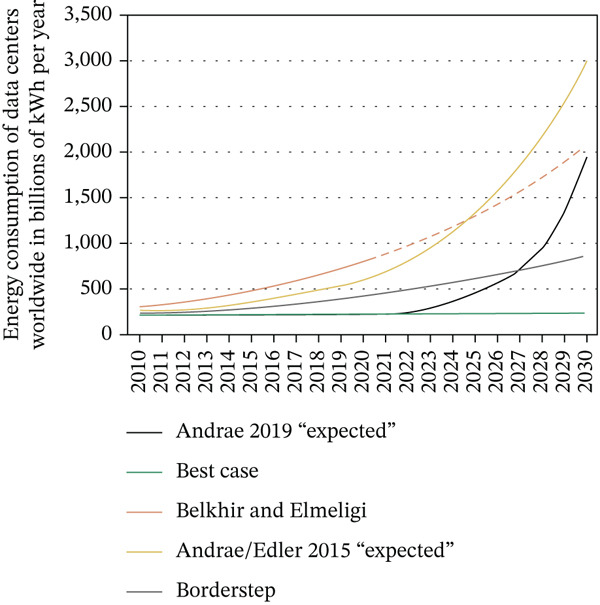
Global server and data center energy usage estimates through 2030.

Since early research, the significance of energy conservation in computing systems has been understood. The overall amount of energy used in DCs has increased despite technological breakthroughs. For instance, DCs′ global energy usage increased by around 115% between 2000 and 2005, from 70.8 to 152.5 TWh. In 2005, this amount accounted for nearly 0.97% of the world′s electricity consumption. With an additional 56% increase in electricity demand between 2005 and 2010, this trend continued [3–5]. DCs used 1.1%–1.5% of the world′s electricity in 2010 alone, with 1.7%–2.2% of the nation′s electricity coming from DCs in the United States. Soaring operating costs, inefficient VM placement, complicated cooling and fire protection demands, and significant ecological impacts particularly with increased greenhouse gas emissions and resource depletion are just a few of the serious issues brought on by DCs′ growing energy consumption. Hardware infrastructure is not the only source of these problems; management practices have a significant impact as well. Leading tech firms like Microsoft, Google, and Amazon have realized this and implemented sophisticated energy‐conscious DC management techniques. Their objective is to reduce energy consumption and environmental impact while increasing return on investment (ROI), maintaining high quality of service (QoS), and guaranteeing adherence to service level agreements (SLAs) [3, 6]. DCs′ energy consumption has skyrocketed due to the quick expansion of cloud computing, creating serious financial and environmental problems. The positioning of VMs on PMs has a significant impact on energy consumption; improper allocation raises carbon emissions and operating expenses [7, 8]. Heuristic algorithms such as first fit decreasing (FFD) and best fit decreasing (BFD) are fast; however, they are not accurate in terms of energy in Figure 1. Despite being more accurate, genetic algorithms (GAs) are slower and require more computing power. Optimizing VM placement is crucial because energy expenditures can make up as much as 80% of DC operating expenses. In order to lower energy consumption and operating expenses in massive virtualized cloud DCs, this research attempts to provide an energy‐efficient VM placement platform using an accelerated GA technique [9, 10].

This report highlights research gaps and recent developments in energy‐efficient VM management in cloud DCs. Due to increasing cloud services, DCs face major operational, financial, and environmental challenges as their energy demand continues to grow beyond existing infrastructure capacity. Energy consumption is influenced by hardware efficiency, workload size, network design, and application requirements. To address this, techniques such as energy‐aware scheduling, virtualization, dynamic resource allocation, VM migration, infrastructure optimization, and autoscaling have been widely studied to build green DCs [11]. However, challenges such as workload imbalance, resource allocation, and QoS requirements still persist. Central processing units (CPUs) are the primary source of power consumption in DCs, and their energy usage depends on utilization and operating frequency. The number of active PMs also significantly affects overall energy use. Traditional methods such as FFD and BFD provide fast allocation but are not energy‐optimal. Many virtualization systems still rely on basic techniques like DVFS and on/off switching rather than intelligent VM placement [12].

Several algorithms have been proposed to minimize the number of active PMs by optimizing VM placement. FFD allocates VMs based on CPU demand but ignores energy efficiency (EE), which can lead to suboptimal resource usage [13]. GAs improve energy‐aware placement but introduce higher computational complexity. In real cloud environments, VMs arrive continuously, requiring dynamic placement and workload profiling. Task classification and prediction improve resource allocation and EE, especially when future workloads are considered. Even small improvements in efficiency can significantly reduce energy cost and environmental impact [14].

The main objectives of this study are as follows:

• To develop an energy‐efficient VM placement model using a GA for cloud DCs.
• To design a lightweight fitness function based on Taylor series approximation that reduces computational complexity from *O*(*G* × *N* × *M* ^2^) to *O*(*G* × *N* × *M*).
• To minimize total energy consumption by reducing the number of active PMs through optimized VM allocation.
• To improve convergence efficiency by reducing execution time and the number of generations required for optimal solutions.
• To enhance resource utilization through workload profiling and task classification (short‐term and long‐term tasks).
• To evaluate and compare the proposed approach with benchmark methods such as FFD and standard GA using real‐world datasets.

This study is organized into five sections. Section 1 presents the background, motivation, and objectives. Section 2 reviews related work, focusing on DCs, GAs, VMs, PMs, EE, and energy optimization (EO). Section 3 describes the proposed methodology and hybrid optimization approach for energy‐aware VM placement. Section 4 presents experimental results and performance comparison with existing methods. Section 5 concludes the study and outlines future research directions in energy‐efficient DC management.

### 1.1. Novelty of the Proposed Work

The novelty of this study is an energy‐efficient GA framework for VM placement in cloud DCs. It introduces a lightweight fitness function based on Taylor series approximation, reducing computational complexity from *O*(*G* × *N* × *M* ^2^) to *O*(*G* × *N* × *M*). The model also integrates workload profiling, task classification (short and long term), and predictive workload handling to improve resource allocation and reduce unnecessary VM migrations. These improvements enhance convergence speed, reduce execution time, and increase EE, making the approach more scalable than existing methods.

## 2. Systematic Literature Review (SLR)

A green or energy‐efficient DC minimizes energy consumption while maintaining performance and reliability. Key metrics include power usage effectiveness (PUE), where values closer to 1 indicate higher efficiency, and carbon usage effectiveness (CUE), which measures carbon emissions per unit of IT energy. Efficient CPU, memory, and storage utilization, along with advanced cooling systems, also reduce energy waste. The use of renewable energy and intelligent workload management, such as energy‐aware VM placement, further improves sustainability.

Recent studies focus on resource management and EO in cloud DCs using techniques like predictive scheduling, GAs, and workload classification. These approaches show that efficient VM placement is essential for improving both performance and energy savings, forming the basis for the proposed workload‐aware optimization model with a lightweight fitness function.

DCs are now an essential component of contemporary infrastructure due to the quick expansion of smart devices, the IoT, and data‐intensive applications. Energy consumption is under increasing strain as Masdari et al. [13] projected that there were over 42.6 billion IoT devices in 2022 and that number is expected to rise to 75.44 billion by 2025. Alharbi et al. [3] stress the significance of energy‐conscious management, application design, and hardware efficiency. Li et al. [15] warn that cost‐effective alternatives could compromise dependability and performance. With servers accounting for over half of all energy consumption, DCs need efficient power management, particularly through virtualization. To ensure sustainability, researchers like Ghribi et al. [16] stress the necessity of continual innovation in VM placement and energy‐efficient resource utilization.

Through DCs, cloud computing provides on‐demand services that make computing resources accessible worldwide. Mercader et al. [17] noted that its objective is to minimize resource consumption while offering elastic, scalable services. To guarantee dependability, Muqaddas et al. [18] stress that major providers like Google, Amazon, and IBM run their global infrastructures under SLAs. Because it enables effective resource sharing and lowers hardware and energy requirements, virtualization is essential. This model reduces computing and data delivery costs, as noted by Moeez et al. [19]. SaaS and IaaS are two types that cloud service providers offer; IaaS allows for on‐demand access to essential computing components. The effect of IaaS is demonstrated by platforms like Google Compute Engine and Amazon EC2. The cornerstone for the deployment of energy‐efficient VMs in cloud settings is IaaS, the subject of this study.

A key component of cloud computing is virtualization, which creates separate VMs to facilitate effective sharing of physical resources. Through resource partitioning, emulation, and time‐sharing, it optimizes system use by abstracting hardware into adaptable, software‐defined units. As in Salahuddin et al. [20], multiple VMs can operate simultaneously on a single physical computer by pooling resources, increasing scalability and flexibility. Because each VM runs independently, Mahmood et al. [21] stated that tasks may be completed safely and effectively. Performance and fault tolerance are enhanced by innovations like virtual storage, networking, and remote access. However, moving VMs can increase energy consumption, which is why energy‐efficient VM placement is being studied. In order to minimize energy consumption and provide QoS, VM allocation seeks to match application requirements with physical resources such as CPU, memory, and bandwidth, as in Hallawi et al. [22].

Effective VM placement in DCs is essential for scalability and energy savings. Zahra et al. [23] stated that underutilized PMs and energy waste are the results of poor placement. Parveen et al. [24] highlighted that strategies seek to save expenses, maximize resources, and limit active PMs. Fast yet inaccurate are early techniques like FFD and BFD, particularly in heterogeneous situations, as highlighted in Afzal et al. [25]. Although more accurate, advanced methods like clustering, GAs, and constraint programming can be computationally expensive. In order to adjust to variations in workload, researchers like Zaman et al. [26] and Tao et al. [27] have also investigated dynamic placement utilizing game theory, evolutionary algorithms, and predictive models. But problems like migration overhead and shifting future demands continue to be major obstacles, as noted by Khan et al. [28].

Large‐scale DCs′ increased energy usage has sparked serious worries. Operating costs are increasing as a result of the global expansion of suppliers like Google and AWS. The electricity consumption of Google′s DCs alone is comparable to that of entire cities. A single DC requires as much electricity as 25,000 houses, according to Khan et al. [29] the increase in nodes in the network can impacts the handling capability of resource consumption, whereas US centers previously relied on 10 nuclear plants for power, according to Khan et al. [30]. According to Gartner, IT energy expenses might increase by 10%–50%virtualization at the IaaS level to, as in Hallawi et al. [22]. Underutilization as a result of overprovisioning for peak loads is a significant problem that results in energy waste, as noted in Laghari et al. [31]. Because electricity is consumed by even inactive servers, according to Khan et al. [32], researchers suggest distribution, load balancing, and optimization; the virtualization at the IaaS level to combine workloads and save energy, promoting greener cloud infrastructures.

Khan et al. [33] used task classification and optimization in industrial operation execution, which helps in categorization techniques in order to enhance algorithm performance and guarantee distribution and balanced load in conventional scheduling algorithms, such as first come first served (FCFS) and shortest job first (SJF). They sought to maximize resource use by matching VM specifications to work size, making sure that VMs for long jobs have better capabilities than those for medium or short activities. But according to the study, more sophisticated methods like GAs or ant colony optimization could improve classification efficacy even more. A complicated optimization model based on multicriteria decision‐making was also proposed by Tao et al. [27], whose framework handles a variety of VM inputs. Heavy workloads necessitate job bundling into VMs prior to mapping them to PMs, according to real‐world statistics from Google′s DCs.

These results demonstrate how efficient job classification can greatly increase cloud DCs′ computational and EE. DCs are using more energy as a result of the increase in VM workloads brought on by the spike in cloud customers. Significant financial gains can result from even modest energy use reductions. Mann and Szabó [8] noted that contextual task information is necessary for practical VM placement solutions because every VM has different requirements. However, the majority of current techniques ignore anticipated workloads and simply address current resource utilization, which frequently results in needless VM migrations.

EE is still a big problem even with cloud computing′s broad usage. Task classification and profiling are essential for DC power consumption reduction and VM placement optimization. DCs can more effectively assign VMs and shut down unused PMs by classifying workloads according to resource requirements. In order to achieve 8%–12% energy savings through profile‐based task grouping, Masdari et al. [13] established a three‐phase methodology for VM placement that uses FFD. Expanding upon this, our research employs a GA to improve VM allocation across time slots, profiling, and categorization. Our GA‐based strategy, in contrast to previous approaches, dynamically optimizes energy use, enabling the shutdown of more PMs and increasing overall efficiency.

By assessing how well each solution satisfies predetermined goals, the fitness function plays a critical role in directing GAs to identify the best options for VM placement. The efficiency of GA can be decreased by local optima caused by poorly constructed fitness functions. According to Khan et al. [34], search efficiency is increased by well‐structured fitness functions, as shown in Table 1.

**Table 1:** Comparative analysis of energy‐efficient VM placement algorithms.

Author(s)	Algorithm	Based on	Resources	Energy efficiency	Migration cost	Computational efficiency
Saxena et al.	SM‐VMP	Genetic algorithm	CPU, memory	?	**✗**	?
Hellmanns et al.	MinPR	Constraint programming	CPU	?	**✗**	**✗**
Gür et al.	ACS	Constraint programming	CPU, memory	?	**✗**	**✗**
Feng et al.	ImmuneGA	Genetic algorithm	CPU, memory, bandwidth	?	**✗**	**✗**
Hallawi et al.	COFFGA and CONFGA	Genetic algorithm and bin packing	CPU, memory	**✗**	?	?
Chang et al.	Hybrid GA	Constraint programming	CPU, memory	?	**✗**	**✗**
Golovin et al.	Min‐Cut	Constraint programming	CPU, network bandwidth	?	**✗**	**✗**
Tao et al.	BGM‐BLA	Genetic algorithm	CPU, network, storage	?	**✗**	**✗**
Jamali et al.	Enhanced FFD	Bin packing	CPU	**✗**	**✗**	?
Gao et al.	VMPACS	Genetic algorithm	CPU, network, storage	?	**✗**	**✗**
Li et al.	EAGLE	Bin packing	CPU, network, storage	?	**✗**	**✗**
Ghribi et al.	Exact allocation and migration	Constraint programming	CPU	?	?	**✗**

To improve speed and accuracy in VM management, this paper proposes a new fitness function that formulates VM placement as a constrained optimization problem. Because fitness evaluation is often the most time‐consuming step in GA, optimizing it can significantly improve overall performance. Recent GA‐based approaches focus on reducing active PMs by efficient workload consolidation and energy‐aware placement. However, challenges still exist, as complex fitness functions often increase computational overhead. Simplifying these processes can improve efficiency without reducing energy performance. VM placement is also difficult due to the large gap between the number of VMs and PMs. Workload classification and profiling help reduce the search space and improve GA efficiency. Although dynamic placement improves flexibility, frequent VM migrations increase energy consumption and reduce performance. Predicting future workloads can reduce unnecessary migrations and support more energy‐efficient VM placement strategies.

## 3. Proposed Methodology

**Figure 2:**
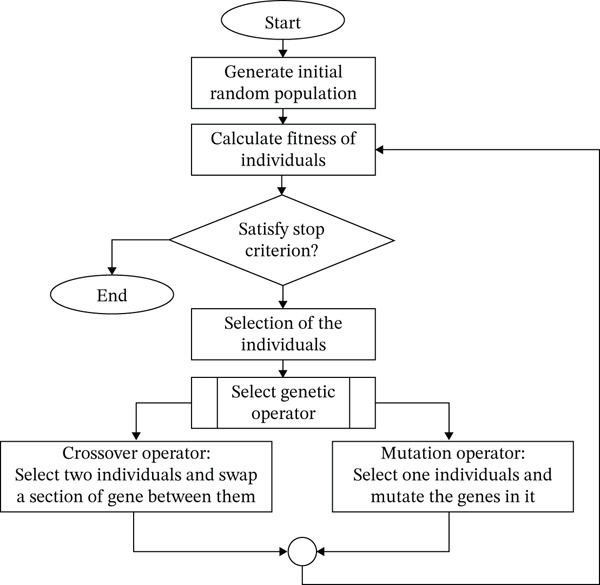
Genetic algorithm flowchart from initialization to termination.

**Figure 3:**
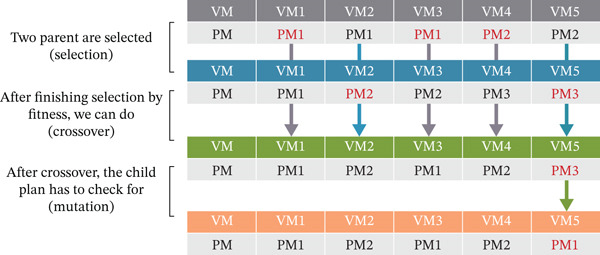
Key genetic algorithm operators demonstrating selection, crossover, and mutation processes.

Exact analytical solutions are frequently impracticable due to the VM placement problem′s NP‐hardness. In order to find the best placement strategies, researchers have therefore resorted to heuristic and meta‐heuristic techniques, including simulated annealing, artificial neural networks, support vector machines, and most famously, GAs. The GA is an attractive option for handling the intricacies of VM allocation because of its versatility and high‐quality solution. By reducing the computational expense usually related to fitness evaluations—which have been found to be the most time‐consuming component of GA operations—this study presents a novel and lightweight fitness function intended to improve GA performance according to Poovizhi et al., even little adjustments can greatly increase overall execution efficiency because the fitness function is assessed several times across generations. The accuracy of the fitness function values assigned to each member of the population is critical to the GA′s performance. The algorithm outperforms benchmark approaches in terms of scalability and adaptability as it iteratively develops, continuously improving the solution space. Figure 2 shows the GA′s general computational flow, and Figure 3 shows its main genetic operators.

### 3.1. GA Operators in VM Placement

In order to improve VM placement algorithms, the GA goes through a number of evolutionary steps:

• Selection: Every member of the present population is assessed for fitness during this phase. People who score higher on fitness are prioritized and kept for the following generation. Stronger candidates are more likely to pass on their traits because each person′s selection probability is calculated using their fitness ratio.
• Crossover: This operator creates new children by fusing the genetic material of two parent solutions, simulating biological reproduction. Crossover adds diversity and enables promising features to be passed down through generations through deliberate recombination.
• Mutation: To explore new regions of the solution space, mutation introduces tiny, arbitrary changes in a person′s chromosomes, expressed as a VM placement plan. In order to preserve genetic variation and avoid early convergence, each gene (which represents the position of a VM and PM) in the progeny has a predetermined probability of being changed.

In order to compare existing solutions to the best one discovered thus far, the algorithm determines each solution′s overall fitness throughout the evaluation phase by evaluating its energy consumption. During the crossover and selection stages, the tournament selection approach is used to strengthen the survival of superior solutions. A VM placement plan, which specifies the particular PM allocated to every incoming VM, is encoded by each individual solution. Throughout the evolutionary process, the algorithm analyzes and continuously assesses these designs′ fitness (EE) in an effort to choose the best configuration.

### 3.2. Data Structure for VM Placement Strategy

**Figure 4:**
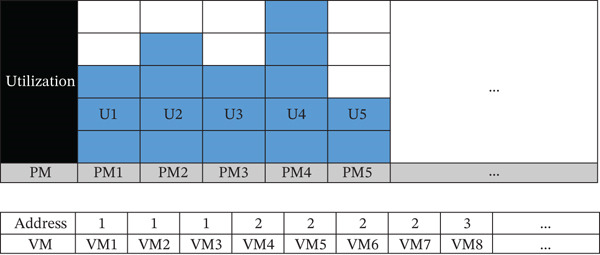
Structured representation of VM–PM mapping and resource allocation.

This study uses the data structure proposed by Ding et al. (2018) for VM placement optimization. The GA relies on this structured representation, which supports fitness evaluation and dynamic VM assignment based on resource availability and constraints. Each solution represents a VM‐to‐PM placement plan, eliminating the need for explicit mapping while tracking resource usage of PMs. The proposed fitness function improves computational efficiency by enabling faster energy‐aware evaluation and faster GA convergence. Workloads are classified using VM, PM, and task profiling to simplify resource allocation and reduce unnecessary computations. The model also considers future task arrivals to improve prediction‐based placement and reduce energy waste. Overall, this structured approach enhances VM placement efficiency and supports an energy‐optimized cloud environment, as shown in Figure 4.

### 3.3. Accelerating GA for Energy‐Efficient VM Placement

The main objective of this work is to accelerate the GA for energy‐efficient VM placement in cloud DCs. A lightweight fitness function is proposed to reduce the computational cost of fitness evaluation, which is the most time‐consuming part of GA. Existing GA approaches improve EE but often suffer from high execution time due to complex fitness calculations. The proposed fitness function improves efficiency while maintaining optimization accuracy by evaluating residual resources of PMs, enabling faster convergence and fewer generations. This leads to reduced execution time and improved energy savings, including better utilization of idle PMs. Experimental results show a 7% reduction in energy consumption compared to FFD and about a 50% reduction in execution time. Unlike previous GA‐based methods, the proposed approach uses a Taylor series–based simplification to reduce complexity from exponential to polynomial time. It also integrates dynamic workload profiling and predictive task grouping into the fitness evaluation process, enabling real‐time energy‐aware VM placement. This combination of lightweight computation and workload intelligence distinguishes the proposed framework from existing approaches.

### 3.4. Efficient Fitness Function Design for GA‐Based VM Placement

**Figure 5:**
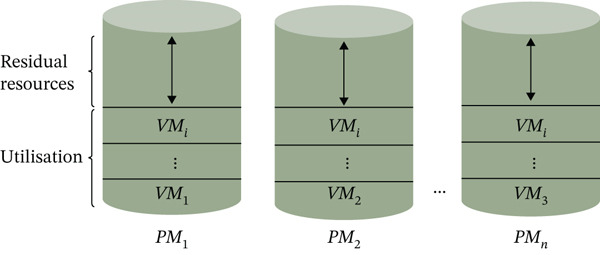
Execution time analysis of fitness function evaluations.

An efficient fitness function is essential for GA to explore large search spaces and identify optimal VM placement solutions. Poorly designed fitness functions can lead to premature convergence or suboptimal results. Therefore, an improved fitness function is proposed that evaluates remaining resources of PMs to enhance EE in cloud DCs. The proposed approach places VMs based on available PM capacity, reducing the number of active PMs and improving resource utilization. This leads to lower energy consumption and faster execution. To further improve efficiency, Taylor series approximation is applied to simplify fitness computation, reducing complexity from exponential to polynomial time and significantly accelerating GA performance, as shown in Figure 5.

An improved fitness function for GA‐based VM placement is proposed for cloud DCs. It first maps each VM to a PM and calculates PM utilization by summing assigned VM resources such as CPU usage. Remaining resources are then computed for each PM, and these values are used in the fitness function to minimize active PMs, improve load balancing, and reduce energy consumption. To demonstrate efficiency, the computational complexity is analyzed using Big‐O notation. Traditional GA‐based methods require *O*(*N* × *M* ^2^) due to pairwise PM comparisons during fitness evaluation. The proposed Taylor series–based approximation reduces this to *O*(*N* × *M*), lowering total GA complexity from *O*(*G* × *N* × *M* ^2^) to *O*(*G* × *N* × *M*). This improvement enhances scalability and significantly reduces execution time, with observed reductions of about 50% in runtime and 48% in convergence generations.

### 3.5. Enhanced Fitness Function Strategy for GA‐Based VM Deployment

In huge search spaces, the GA must be guided toward optimal solutions via an efficient fitness function. Each potential answer is given a score determined by how appropriate it is. Whereas well‐designed fitness functions promote exploration, poorly constructed ones can trap the GA in local optima, as noted in Khan et al. [34]. According to Rao et al., we develop a unique fitness function that makes use of the leftover resources of PMs in order to address EE in cloud DCs. This method boosts energy savings, speeds up execution, and lowers active PMs. Figure 3 illustrates that although the number of active PMs declines as residual resources are effectively managed, PM resource usage increases with VM load. We use the Taylor expansion, a mathematical tool that uses polynomial approximations to simplify difficult operations, to further increase efficiency. The fitness evaluation is then converted from exponential to polynomial time.

The improved method for determining the fitness value in a GA‐based VM deployment strategy for cloud DCs is described in the following steps:

• Go over each VM in the given deployment plan one by one.
• Determine which PM the VM is assigned to.
• Add the VM′s resource requirements (such as CPU consumption) to the PM′s resource utilization.
• If the PM was not active before, activate it.
• Finish the VM‐to‐PM mapping process.
• Set the counter for the total residual resources to zero.
• Go through every PM that is currently live.
• Verify whether the PM is hosting any VMs (i.e., whether usage > 0).
• Subtract the used resources from the PM′s total capacity to determine its remaining resources.
• To the total residual resource counter, add the residual value.
• Put a stop to the conditional check.
• Complete the iteration of PM.
• Provide the final fitness value, which is the sum of the remaining resources for all active PMs.

This experiment′s main goal is to assess how well the suggested fitness function works with a GA to optimize VM placement. We run extensive simulation experiments using the increased fitness function and examine a number of important metrics to illustrate its performance. These include the plan for assigning applications, the VM placement strategy that results, the number of PMs that must be in use at any given time in order to support all task‐loaded VMs, the DC′s overall power consumption during the evaluation period, and the time needed to calculate virtual resource allocation. A variety of DC environments, including small‐, medium‐, and large‐scale infrastructures, are covered by our simulations. We can evaluate the suggested approach′s scalability, efficiency, and flexibility under different workload intensities and resource limits thanks to this multiscale evaluation.

### 3.6. Cloud‐Based Dataset

We use the openly accessible Google cluster‐usage traces from Jamali and Malektaji [35] to simulate computational workloads in order to illustrate our VM placement method. These traces are commonly accepted as a trustworthy standard for assessing resource management tactics in cloud environments, and they come from a sizable, nonvirtualized DC. Researchers are urged to utilize these traces to build and test simulation studies, as they contain extensive real‐world data including specific task settings, resource requests, and actual allocations, as shown in Table 2. The dataset includes 5‐min interval recordings of DC activities over the course of a full month. To preserve computational viability and guarantee accurate workload representation, we limit our simulation to a 24‐h subset. Incoming tasks (workload) are first mapped to VMs and subsequently assigned to PMs for execution within each 5‐min timeframe. The particular workload configurations for the small, medium, and large DC scales are shown in Table 3. The efficiency of the fitness function may be fairly compared across various operational scales thanks to this configuration.

**Table 2:** Setup details of cloud‐based data center scenarios (benchmark values).

Dataset	Small (S)	Medium (M)	Large (L)
Task (VMs)	1350	2242	5365
PMs	1000	2000	3000

**Table 3:** Setup details of cloud‐based data center scenarios (standard benchmark conventions).

Variables	Range
Size of population	64
Tournament size (for selection)	16
Elitism	True
Uniform rate (for crossover)	0.5
Rate of mutation	0.015
Run	50
Max iterations	999

### 3.7. GA Configuration and Parameters

Although our research does not primarily focus on parameter adjustment, we used recognized standard settings to guarantee consistent and dependable GA performance. Our GA made use of a 64‐person population, which is a widely used size that strikes a compromise between exploration and convergence effectiveness. Smaller populations run the risk of prematurely stagnating in local optima, even if they might result in faster convergence. Larger populations, on the other hand, offer greater diversity but come at the expense of longer processing time. We used elitism, which keeps the top performers across generations, to improve convergence without sacrificing diversity. This method helped down the overall number of generations needed by applying Alharbi et al.′s FFD heuristic [3]. However, elitism had no direct impact on fitness score computations throughout our simulations because the fitness evaluation was done consistently to all participants based on VM placement plans. We used the tournament selection technique put forward by Jebari et al. [48] as the selection process. In order to provide a competitive yet diverse selection procedure, the tournament pool was first randomized and then later resized to 16. In accordance, we used an adaptive mutation technique with a low mutation rate of 0.015 to avoid premature convergence or needless delays. In order to ensure computational efficiency and prevent overfitting or stagnation, the GA was finally stopped after 50 consecutive generations if no discernible improvement was seen.

### 3.8. Experimental Setup

A desktop computer with an Intel Core i7‐8650U CPU running at 2.11 GHz and 16 GB of DDR4 RAM (2666 MHz) was used in a series of simulation experiments to assess the efficacy of the suggested fitness function for GA‐based VM placement in cloud DCs. The simulation program was created using Java JDK 1.8.0_171, and the simulation environment was configured on Windows 10 Professional using Eclipse Neon.4 as the integrated development environment (IDE). The simulation models one‐time incoming workloads using real‐world task data taken from Google′s cluster traces. Algorithm 2, which manages the mapping of computing jobs to VMs based on their resource demands, is used to initially assign these tasks to VMs. Our suggested GA‐based fitness function is used to optimize the VMs′ distribution among PMs after they have been assigned. The evaluation′s findings are presented as the average of several simulation runs with different DC sizes. These findings shed light on how well the proposed solution performs in terms of overall system responsiveness, energy usage, and VM allocation efficiency.

## 4. Results and Discussion

The computing efficiency of the proposed approach was evaluated using multiple performance metrics, including fitness evaluation time, total execution time of the GA, number of generations required for convergence, and average time per generation. These metrics provide a comprehensive assessment of computational performance. The proposed fitness function was compared against the standard GA and FFD heuristic under identical experimental conditions across small‐, medium‐, and large‐scale datasets. Efficiency improvement was quantified based on percentage reduction in execution time and number of iterations required to reach convergence. Additionally, scalability was analyzed by observing performance trends with increasing workload size. The results demonstrate that the proposed method significantly enhances computational efficiency while maintaining EO performance.

The evaluation outcomes of proposed studies on small‐, medium‐, and large‐scale datasets are shown in this section. While maintaining constant settings across all scales (with the exception of input size), the study evaluated the performance of the suggested method in comparison to industry‐standard techniques like the FFD heuristic and the conventional GA (standard GA). The method utilizing a simple energy‐based fitness function is referred to as “standard GA” in the visual results, but “our GA” integrates an enhanced fitness function intended to improve EE in VM placement. In this study, instantaneous power measurements (W) were not considered as the primary evaluation metric because they represent momentary snapshots of energy usage and do not reflect the cumulative operational cost of cloud DC workloads. Instead, energy consumption (kWh) was used as the evaluation metric, as it provides a time‐integrated measure of power usage and is widely adopted in cloud computing and DC optimization studies. Because VM placement decisions and workload execution occur over time intervals, energy consumption offers a more meaningful and realistic representation of system performance. Instantaneous power values can fluctuate significantly due to dynamic workload variations, making them less reliable for comparative analysis across different algorithms. Therefore, integrating power over time to compute total energy consumption ensures a fair and consistent evaluation of the proposed GA‐based optimization approach. This approach is aligned with standard practices in energy‐aware cloud computing research.

The power consumption values presented in Table 4 are based on standard benchmark assumptions commonly used in cloud and edge computing simulation studies. These values represent relative energy consumption levels across different computing layers, where the cloud DC exhibits the highest power usage due to its large‐scale infrastructure and continuous operation, whereas edge nodes consume comparatively lower power due to their limited computational capacity. The selected values (60 W for cloud and 20 and 10 W for edge nodes) are adopted to reflect realistic proportional differences in energy consumption and to enable consistent comparative evaluation of the proposed optimization approach. Such normalized configurations are widely used in simulation‐based research to analyze system performance under controlled conditions.

**Table 4:** Optimized energy consumption metrics for task offloading in IoT systems.

Component	Power consumption
Cloud data center	60 W
Edge node 1	20 W
Edge node 2	10 W

The regular GA, on the other hand, maximizes CPU utilization per PM in order to achieve EE, as shown in Table 5. The outcomes show that our approach not only accomplishes effective energy‐aware VM placement but also cuts down on calculation time.

**Table 5:** Fitness time (s) comparison between standard and proposed fitness functions.

Fitness function	Large tasks	Medium tasks	Small tasks
Standard fitness function	569.1	201.9	102.4
Proposed fitness function	235.9	115.9	78.5

**Figure 6:**
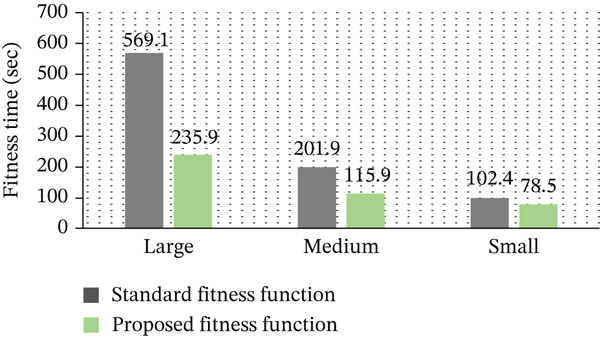
Execution time comparison of fitness evaluation strategies.

Figure 6 presents the comparison of fitness evaluation time between the standard GA and the proposed fitness function across different dataset sizes. The results clearly show that the proposed approach significantly reduces the fitness computation time. This improvement is primarily due to the use of the Taylor series–based simplification, which reduces the computational complexity from quadratic to linear. Consequently, faster fitness evaluation contributes to overall reduction in GA execution time and enhances scalability for large‐scale DC environments.

The main goal is to speed up the GA′s overall performance by cutting down on the amount of time needed to calculate the fitness function. When compared to the conventional GA, our suggested fitness function significantly reduces calculation time by up to 59%, resulting in a more effective evaluation procedure. Our GA converges more quickly than conventional methods thanks to this enhancement, which makes it possible for the population′s average fitness value to rise more quickly inside each generation, as shown in Table 6.

**Table 6:** Execution time of genetic algorithm (GA) using standard versus proposed fitness functions.

Fitness function	Large tasks (s)	Medium tasks (s)	Small tasks (s)
Standard fitness function	819.3	258.9	141.5
Proposed fitness function	411.4	146.5	97.3

**Figure 7:**
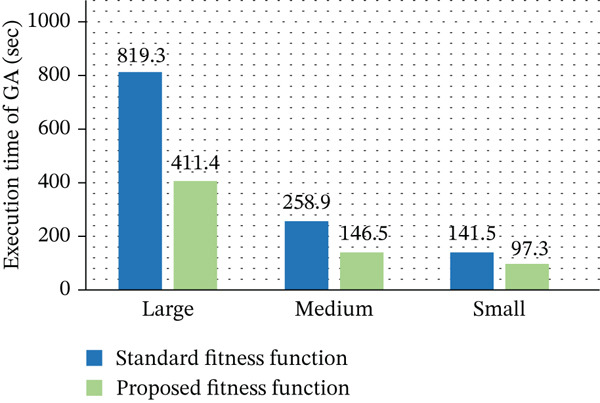
Average execution time analysis of genetic algorithm.

Figure 7 illustrates the average execution time of the GA using both the standard and proposed fitness functions. The results clearly indicate that the proposed approach significantly reduces execution time across all dataset sizes. This improvement is achieved due to the simplified fitness evaluation using the Taylor series approximation, which lowers computational complexity and accelerates convergence. Furthermore, the suggested fitness function effectively increases speed and scalability by reducing the GA′s overall execution time by 50% on large‐scale datasets.

**Figure 8:**
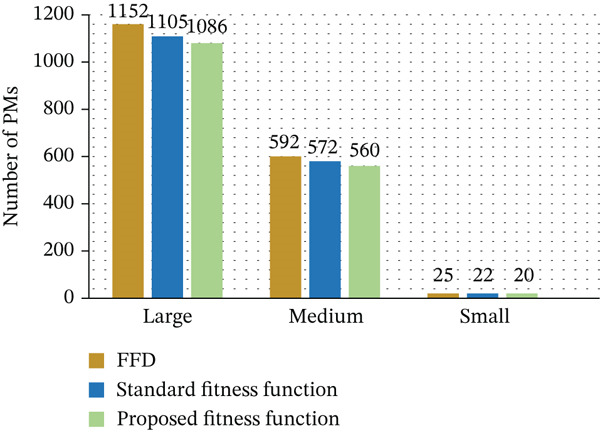
Optimized PM utilization in cloud data centers.

Figure 8 demonstrates the optimized utilization of PMs in the proposed framework. The results show that fewer PMs are required to handle the same workload, indicating improved resource utilization. This reduction is achieved through efficient VM placement and workload consolidation, which directly contributes to lower energy consumption. The outcomes verify that the platform′s optimization successfully utilizes PM consumption to carry out the fitness function more effectively. We concentrate on lowering the number of generations needed for convergence in order to improve the GA′s EE, as discussed in Table 7. We successfully reduce the search space by tightening limitations, which results in fewer generations.

**Table 7:** Number of generations: Standard versus proposed approaches.

Approach	Large tasks	Medium tasks	Small tasks
Standard	355	234	174
Proposed	184	147	128

**Figure 9:**
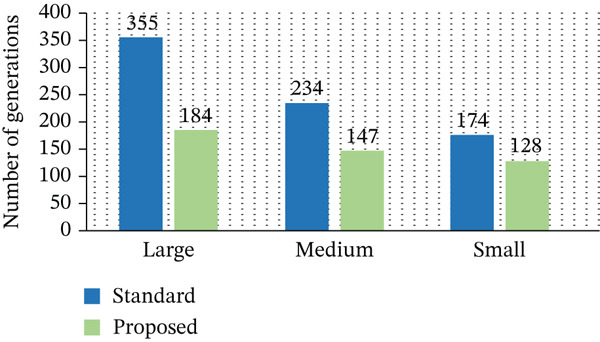
Mean execution time per generation in GA.

Figure 9 shows the number of generations required for convergence in both the standard and proposed GA approaches. The proposed method significantly reduces the number of generations needed to reach optimal solutions. This is mainly due to the efficient fitness function design, which guides the search process more effectively and reduces unnecessary iterations. As the chart makes evident, this notable improvement demonstrates the effectiveness of our energy‐optimized VM placement technique, which continuously surpasses the conventional GA in terms of speed and performance, as discussed in Table 8.

**Table 8:** Generation duration (s): Standard versus proposed fitness functions.

Fitness function	Large tasks (s)	Medium tasks (s)	Small tasks (s)
Standard fitness function	3.28	1.38	0.89
Proposed fitness function	2.23	1.11	0.64

**Figure 10:**
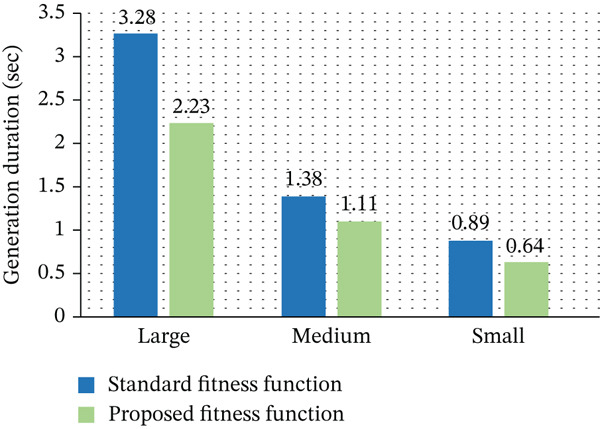
Average time per generation using GA variants.

Figure 10 presents the average time required per generation for both GA variants. The proposed approach consistently demonstrates lower computation time per generation, highlighting its efficiency. This improvement results from reduced complexity in fitness evaluation, enabling faster processing of candidate solutions. Using our suggested fitness function, the average amount of time spent by the GA every generation across three distinct dataset scales is provided. The results demonstrate that our approach cuts generation time by 32% when compared to the usual GA, which assesses fitness in each generation. EE in this study is measured using the total energy consumed by all active PMs during VM execution. It is computed by summing the energy usage of each PM based on its CPU utilization over the simulation period. Lower total energy consumption indicates higher EE. The proposed GA improves efficiency by reducing the number of active PMs and optimizing VM‐to‐PM allocation, leading to reduced overall energy usage compared to baseline methods.

Large‐scale DCs have significant energy requirements; therefore, this enhancement signifies a significant drop in operating expenses. This study′s main contribution is the introduction of a unique fitness function that maintains excellent energy‐saving performance while also increasing the GA′s computing efficiency, as discussed in Table 9. To provide consistency among all the experimental representations, all energy values appearing in tables and figures are normalized to kWh units of energy that represent total DC energy consumption during each evaluation period. Power consumption values given in watts were normalized into kWh units of energy by integrating power over time intervals; hence, such data are comparable with cumulative energy values. The percentage values correspond to relative enhancements or savings with respect to the baseline FFD approach. All the units and numerical values were checked for consistency, and figures were labeled accordingly to reflect total DC energy consumption instead of instantaneous power measurements.

**Table 9:** Energy consumption (kWh): FFD versus standard and proposed fitness functions.

Task size	FFD	Standard fitness function	Proposed fitness function
Large	617	578	570
Medium	396	303	294
Small	122	116	115

**Figure 11:**
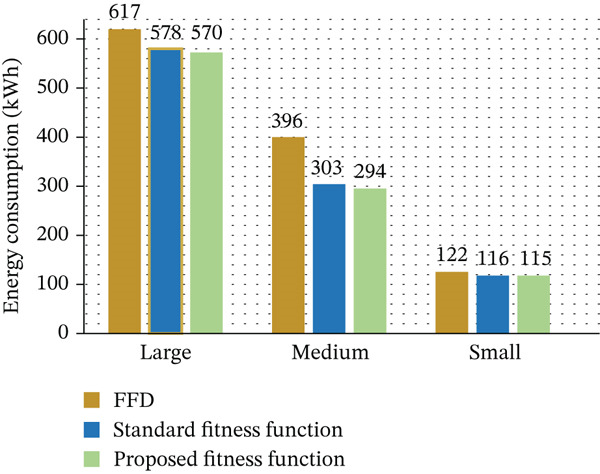
Energy consumption per time slot with and without task classification.

As shown in Figure 11, the analysis validates that our suggested VM allocation technique dramatically lowers energy consumption in cloud DCs. Conventional power‐based fitness evaluation is the foundation of the traditional method, often known as the standard GA. Our GA, on the other hand, improves EE by using a fitness function based on the Taylor expansion. We compared our method to the FFD algorithm and the conventional GA. In comparison to FFD, our approach uses around 7% less energy, as seen in Figure 11.

### 4.1. Environmental Impact

The proposed study develops energy‐based evaluation by quantifying the corresponding carbon footprint. Carbon emissions (CO_2_e) are estimated by using a standardized emission factor to transform energy consumption in kWh from DCs into CO_2_ equivalents, converted using the following formula:CO_2_e (kg) = energy consumption (kWh) × emission factor (kg CO_2_e/kWh)

An average emission factor of 0.475 kg CO_2_e/kWh is used, by following US EPA (Environmental Protection Agency) guidelines, which represents global grid electricity averages. Using this mapping, the proposed GA‐based model reaches an energy consumption of around 7% less and a corresponding reduction of 6.8% in CO_2_ emissions compared to the FFD method. For example, a demand for 570 kWh of energy translates into 270.75 kg CO_2_e, whereas the baseline FFD amounts to 617 kWh, which is equal to 293.08 kg CO_2_e, thus representing an environmental benefit of the proposed optimization approach. Results at this quantitative level consolidate the claim concerning a reduction of carbon emissions and strengthen the connection between EE and sustainable operation of DCs.

### 4.2. Task Classification Impact on EE and Execution Time in GA‐Based VM Placement

**Figure 12:**
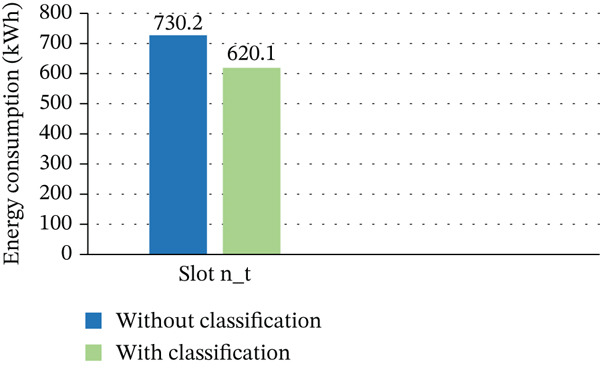
Energy consumption per time slot with and without task classification.

The results of all pertinent studies, both with and without task categorization in cloud DCs, are shown in this section. Implementing measures that reduce usage of electricity has become crucial due to the growing concern over rising energy consumption. Our strategy emphasizes resource profiling and workload classification to improve energy‐efficient VM deployment. We make it possible to allocate resources more intelligently by dividing workloads into short‐term and long‐term activities and allocating them to the appropriate VMs. PMs, VMs, and profiling activities all contribute to precise resource availability assessment and performance optimization over time. This classification‐driven architecture greatly increases the energy efficacy of the DC operations by providing a basis for power‐aware VM placement. According to the study, cloud DCs′ EE is greatly increased when task classification is incorporated into VM distribution. Our task‐aware method, which is based on an accelerated GA, reduces energy usage by 15% when compared to the same GA without classification, as shown in Figure 12. To do this, workloads in each time slot are separated into short‐term and long‐term tasks, enabling more focused energy usage calculations and smarter VM deployment. The findings demonstrate how workload classification can facilitate more intelligent, energy‐efficient resource management and enhanced overall performance in dynamic cloud environments, as discussed in Table 10.

**Table 10:** Energy consumption (kWh) with and without classification.

	Without classification	With classification
Slot n_t	730.2	620.1

**Figure 13:**
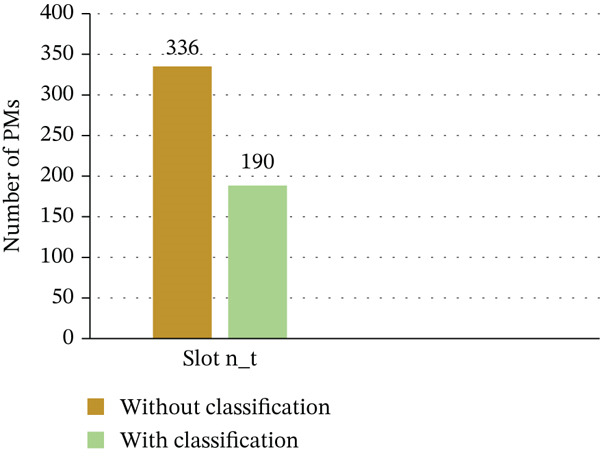
Impact of task classification on PM utilization.

The efficiency of our accelerated GA, driven by the suggested optimization platform, in reducing the quantity of running PMs in a cloud DC is demonstrated in Figure 13. A key component of this improvement is task classification. There is a notable drop in PM usage when comparing the GA with and without task categorization; there are roughly 146 less active PMs during peak hours, which represents a 43% decrease. Furthermore, according to our suggested fitness function, the accelerated GA continuously uses fewer PMs, resulting in a 15% decrease in energy consumption each time slot—from 730 to 620 kWh. These results demonstrate the method′s great potential to improve cloud environments′ sustainable energy management and resource efficiency, as discussed in Table 11.

**Table 11:** Number of physical machines (PMs) with and without classification for slot n_t.

Classification type	Number of PMs
Without classification	336
With classification	190

**Figure 14:**
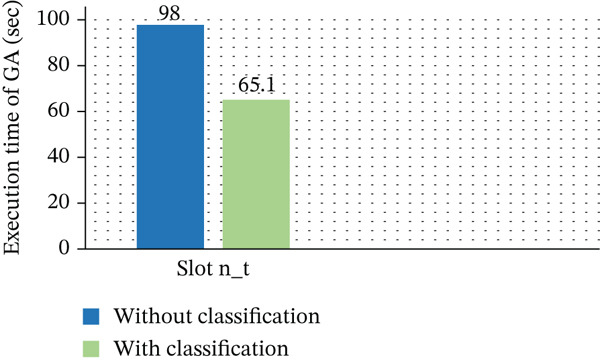
Execution time comparison of GA with and without task classification.

The overall execution time of our suggested GA on large‐scale datasets, both with and without task classification, is shown in Figure 14. The method greatly lowers computational burden and increases processing performance by implementing a task‐aware fitness function. By separating tasks into short‐term and long‐term categories, it is possible to allocate VMs independently and prevent the inefficiencies that come with processing all VMs at once. Its efficiency and scalability are highlighted by the fact that task categorization reduces execution time by 33% as compared to the nonclassified GA. By matching appropriate VMs with high‐intensity workloads, this approach enhances throughput and responsiveness. The task‐aware GA attains a good trade‐off between speed and power efficiency, although introducing a slight increase in energy consumption. For optimal cloud DC performance, these findings support the importance of incorporating workload classification into GA‐based VM placement frameworks, as discussed in Table 12.

**Table 12:** Execution time of genetic algorithm (GA) with and without classification.

	Execution time without classification (s)	Execution time with classification (s)
Slot n_t	98	65.1

## 5. Comparative Analysis

The most recent research contributions on energy‐efficient VM placement in cloud DCs are compiled in this comparative analysis table, which highlights various approaches meant to lower power consumption and maximize resource usage. Strategic energy reduction is emphasized by Hallawi et al. [22] and Ding et al. [7], the latter of whom advocate a comprehensive, multilayered optimization method. Tao et al. [27] used real‐world data for multicriteria optimization, whereas Masdari et al. [13] provided a static model based on profiling, which resulted in quantifiable energy savings. Khan et al. [28] used predictive strategies to address workload variability, whereas Khan et al. [33] improved traditional scheduling through job classification. These studies still confront obstacles such as system complexities, limited adaptability, and migrating overhead despite their progress. The goal of our proposed study, on the other hand, is to increase energy economy and scalability through intelligent VM allocation across time slots by introducing a dynamic, GA‐based model that incorporates workload profiling and a bespoke fitness function, as discussed in Table 13.

**Table 13:** Comparative analysis of recent advancements in energy‐efficient VM placement techniques.

Author(s)	Focus area	Technique/model used	Key contribution	Limitation/challenge
Hallawi et al. [22]	Strategies for energy reduction	Placement of VMs and power management	Highlighted efficient energy‐saving techniques for any virtual machine allocation	Underutilization as a result of high supply
Ding et al. [7]	Comprehensive optimization	Optimization of hardware, resources, and applications	Suggested a framework for three‐level optimization.	Scalability and difficulty of integration
Masdari et al. [13]	Organizing tasks to save energy	FFD‐based three‐phase model	8%–12% energy savings were attained using task profiling.	Limitations of FFD and static allocation
Tao et al. [27]	Optimization based on multiple criteria	Smart placement of virtual machines	Suggested optimization model with actual Google data	Complexity and managing a heavy workload
Khan et al. [33]	VM scheduling and classification	SJF and FCFS with task categorization	VM specifications were matched to workload types, increasing utilization.	GAs and ACO recommendations for improved outcomes
Khan et al. [28]	Adaptive workload management	Models for prediction and game theory	Used adaptable positioning to address varying demands.	Forecasting accuracy and migration overhead
Our proposed work	Allocation of resources with low energy consumption	GA with task profiling and custom fitness function	Workload‐aware GA for dynamic virtual machine allocation across time slots	Awaiting benchmarks and real‐world deployment

## 6. Conclusion

DCs are a fundamental component of modern cloud computing infrastructure, and their rapid expansion—driven by increasing internet traffic, IoT, and data‐intensive applications [36, 37, 38]—has resulted in significant energy consumption and rising operational costs [39, 40, 41]. This study addressed these challenges by proposing an energy‐efficient VM placement framework based on an improved GA. The proposed method introduces a lightweight fitness function using Taylor series approximation, which significantly reduces computational complexity and improves convergence efficiency [42, 43, 44, 45]. Experimental results demonstrate that the proposed approach reduces energy consumption by approximately 7% compared to the FFD method, decreases execution time by up to 40%, and reduces the number of generations required for convergence by around 48%. Furthermore, improved workload profiling and task classification enable better resource utilization, resulting in reduced activation of PMs and enhanced system efficiency. Overall, the proposed framework successfully achieves a balance between EE and QoS requirements in cloud DCs. The results confirm that combining intelligent optimization techniques with lightweight fitness evaluation can significantly enhance scalability and sustainability in cloud environments.

## 7. Limitations and Future Directions

Although the proposed approach demonstrates strong performance in energy‐efficient VM placement, certain limitations remain. The current model does not fully incorporate dynamic and highly unpredictable workload arrivals, which may affect real‐time decision‐making accuracy. Additionally, application‐level behavior profiling is limited and can be further enhanced to improve resource prediction and allocation efficiency. Future work will focus on integrating predictive machine learning models to handle dynamic workloads more effectively and extending the framework to multiresource optimization involving CPU, memory, storage, and network. Moreover, large‐scale real‐world validation using platforms such as OpenStack or VMware is recommended to further evaluate system robustness and practical applicability. These improvements will contribute toward the development of fully adaptive, intelligent, and sustainable cloud DC architectures.

**Nomenclature**
ACS: ant colony system
BFD: best fit decreasing
CO_2_
: carbon dioxide
CO_2_e: carbon dioxide equivalent
CPU: central processing unit
DC: data center
DVFS: dynamic voltage and frequency scaling
EE: energy efficiency
EO: energy optimization
FFD: first fit decreasing
GA: genetic algorithm
IaaS: infrastructure as a service
IoT: Internet of Things
kWh: kilowatt‐hour
PM: physical machine
QoS: quality of service
SLA: service level agreement
SLR: systematic literature review
VM: virtual machine

## Author Contributions

Yasir Afzal and Naila Nawaz perform the original writing part, software, and methodology. Yasir Afzal, Naila Nawaz, Abdullah Ayub Khan, Muhammad Jawad Yousaf, Muhammad Zohaib Khan, Erssa Arif, Muqaddas Salahuddin, Mohamad Afendee Mohamed, and Sajid Ullah perform rewriting, investigation, design methodology, and conceptualization. Yasir Afzal, Naila Nawaz, Abdullah Ayub Khan, Muhammad Jawad Yousaf, Muhammad Zohaib Khan, Erssa Arif, Muqaddas Salahuddin, Mohamad Afendee Mohamed, and Sajid Ullah perform related work part and manage results and discussions. Yasir Afzal, Naila Nawaz, Abdullah Ayub Khan, Muhammad Jawad Yousaf, Muhammad Zohaib Khan, Erssa Arif, Muqaddas Salahuddin, Mohamad Afendee Mohamed, and Sajid Ullah perform rewriting, design methodology, and visualization.

## Funding

No funding was received for this manuscript.

## Disclosure

The author accepts the online/published version of this manuscript.

## Ethics Statement

The committee of the University of Sultan Zainal Abidin Malaysia confirmed that all experimental protocols were approved by the organization. It is confirmed that the experiments follow the criteria of ethics approval and consent to participate.

## Consent

No human subjects are harmed in this research, and we confirmed that all data are shared with the participants.

## Conflicts of Interest

The authors declare no conflicts of interest.

## Data Availability

The data that support the findings of this study are available from the corresponding author upon reasonable request.
